# Blood pressure in adolescents and young adults with type 1 diabetes: data from the Australasian Diabetes Data Network registry

**DOI:** 10.1007/s00592-023-02057-4

**Published:** 2023-03-15

**Authors:** Steven James, Lin Perry, Julia Lowe, Margaret Harris, Peter G. Colman, Maria E. Craig, Australasian Diabetes Data Network Study Group, Kym Anderson, Sof Andrikopoulos, Geoff Ambler, Helen Barrett, Jenny Batch, Philip Bergman, Fergus Cameron, Louise Conwell, Andrew Cotterill, Chris Cooper, Jennifer Couper, Elizabeth Davis, Martin de Bock, Kim Donaghue, Jan Fairchild, Gerry Fegan, Spiros Fourlanos, Sarah Glastras, Peter Goss, Leonie Gray, Peter Shane Hamblin, Paul Hofman, Dianne Jane Holmes-Walker, Tony Huynh, Sonia Isaacs, Craig Jefferies, Stephanie Johnson, Tim Jones, Jeff Kao, Bruce R. King, Antony Lafferty, Jane Makin, Michelle Martin, Robert McCrossin, Kris Neville, Mark Pascoe, Ryan Paul, Dorota Pawlak, Alexia Peña, Liza Phillips, Darrell Price, Christine Rodda, David Simmons, Richard Sinnott, Carmel Smart, Anthony Stell, Monique Stone, Steve Stranks, Elaine Tham, Barbara Waddell, Glenn Ward, Ben Wheeler, Helen Woodhead, Anthony Zimmermann

**Affiliations:** 1 School of Nursing, Midwifery and Paramedicine, University of the Sunshine Coast, 1 Moreton Parade, Petrie, 4502 Australia; 2 Medicine, Dentistry and Health Sciences, University of Melbourne, Parkville, 3010 Australia; 3 School of Nursing and Midwifery, University of Technology Sydney, Ultimo, 2007 Australia; 4 Nursing Research and Practice Development, Prince of Wales Hospital, Randwick, 2031 Australia; 5 Department of Medicine, University of Toronto, Toronto, M5S 1A8 Canada; 6 School of Nursing and Midwifery, University of Newcastle, Callaghan, 2308 Australia; 7 Diabetes and Endocrinology, The Royal Melbourne Hospital, Parkville, 3050 Australia; 8 Endocrinology, Children’s Hospital at Westmead, Westmead, 2145 Australia; 9 Faculty of Medicine and Health, University of Sydney, Camperdown, 2006 Australia; 10 School of Clinical Medicine, University of New South Wales, Kensington, 2033 Australia

**Keywords:** Adolescents, Blood pressure, Hypertension, Type 1 diabetes, Young adults

## Abstract

**Aim:**

Hypertension increases complication risk in type 1 diabetes (T1D). We examined blood pressure (BP) in adolescents and young adults with T1D from the Australasian Diabetes Data Network, a prospective clinical diabetes registry in Australia and New Zealand.

**Methods:**

This was a longitudinal study of prospectively collected registry data. Inclusion criteria: T1D (duration ≥ 1 year) and age 16–25 years at last visit (2011–2020). Hypertension was defined as (on ≥ 3 occasions) systolic BP and/or diastolic BP > 95^th^ percentile for age < 18 years, and systolic BP > 130 and/or diastolic BP > 80 mmHg for age ≥ 18 years. Multivariable Generalised Estimating Equations were used to examine demographic and clinical factors associated with BP in the hypertensive range across all visits.

**Results:**

Data from 6338 young people (male 52.6%) attending 24 participating centres across 36,655 T1D healthcare visits were included; 2812 (44.4%) had BP recorded at last visit. Across all visits, 19.4% of youth aged < 18 years and 21.7% of those aged ≥ 18 years met criteria for hypertension. In both age groups, BP in the hypertensive range was associated with male sex, injection (vs. pump) therapy, higher HbA1c, and higher body mass index.

**Conclusions:**

There is a high proportion of adolescents and young adults reported with BP persistently in hypertensive ranges. Findings flag the additive contribution of hypertension to the well-established body of evidence indicating a need to review healthcare models for adolescents and young adults with T1D.

## Introduction

The incidence of type 1 diabetes (T1D) is increasing worldwide, especially in children and young people [1–3]. This is particularly concerning since people diagnosed with T1D at age < 30 years have up to five-fold excess mortality risk [4]. The major causes of premature mortality are vascular complications, aggravated through co-morbid diseases such as hypertension [5, 6]. Linked to peripheral, cerebro- and cardiovascular disease, the sequelae of hypertension may include limb amputations, stroke, cardiac failure and sudden death. The risks of these increase with longer duration of hypertension, especially when uncontrolled.

Clinical practice guidelines for children and adolescents define hypertension as a systolic and/or diastolic blood pressure (BP) that is ≥ 95th percentile for sex, age and height on ≥ 3 occasions [7]. For adults, hypertension is defined as a sustained BP ≥ 140/90 mmHg, with lower systolic and diastolic BP targets appropriate for individuals at high risk of cardiovascular disease if they can be achieved without undue treatment burden [8]. Australian clinical care guidelines for T1D recommend BP < 130/80 mmHg in adults, and < 125/75 mmHg in the presence of ≥ 1 g of proteinuria per day [9]. We previously reported that hypertension occurred internationally in almost 50% of young adults with T1D [5]; however there are otherwise limited data in young people with T1D in Australasia [10–12]. More detailed data are needed to inform healthcare, to prevent and treat hypertension in this population.

Using data from the Australasian Diabetes Data Network (ADDN), a prospective clinical diabetes registry established in 2012 [13, 14], we examined BP in adolescents and young adults with T1D across Australia and New Zealand, and examined factors associated with BP in the hypertensive range in this population.

## Methods

### Design

This was a longitudinal study of prospectively collected registry data.

### Population

Clinical data in ADDN were prospectively collected from 24 participating centres across Australia and New Zealand, of which 13 (54.2%) were paediatric centres. We included individuals with T1D duration ≥ 1 year who were aged 16–25 years at their last recorded T1D healthcare visit (between 1st January 2011 and 31st December 2020). This age range was chosen since adolescence is recognised as a distinct phase of maturation, variably but generally complete by age 25 years [15]. Data extracted comprised demographic and clinical variables, including date of birth, sex, number of visits, age at T1D diagnosis, insulin regimen, BP, height, weight and body mass index (BMI).

### Definitions and approvals

BP was defined as being in hypertensive ranges when participants aged < 18 years had a systolic and/or diastolic BP at ≥ 95th percentile and, for those aged ≥ 18 years, systolic BP ≥ 130 and/or diastolic BP ≥ 80 mmHg [16]. For those aged < 18 years, standardised BMI scores were based on CDC reference data [17]. Overweight/obesity was defined as BMI standard deviation score (SDS) ≥ 85th percentile for those aged < 18 years or BMI > 25 kg/m^2^ for those ≥ 18 years. All centres had Human Research or Health and Disability Ethics Committee approval for participation in ADDN, and the current study was approved by the University of the Sunshine Coast Human Research Ethics Committee, Australia (reference: E19113).

### Statistical methods

Descriptive statistics are reported as mean ± standard deviation for parametric data. The primary outcome was BP in the hypertensive range. Univariate associations between categorical variables were examined using Chi-square tests and for continuous variables using *t*-tests. Hypertensive range BP measurements were stratified by HbA1c and sex, as previous studies have emphasised the impact of these parameters [18–21]. Multivariable generalised estimating equation (GEE) models were used to examine factors associated with BP in the hypertensive range across all visits, with explanatory variables in the models including T1D duration, sex, T1D therapy (use of twice daily (BD)/multiple daily injections (MDI) vs. continuous subcutaneous insulin infusion therapy (CSII)), HbA1c and BMI; variables chosen based on focused on clinical knowledge and previous literature. Goodness of fit was assessed using the two extensions of Akaike’s information criterion for model selection: quasi-likelihood under the independence model criterion (QIC) for choosing the best correlation structure and another QIC measure for choosing the best subset of predictors. Results are reported as beta and 95% confidence intervals (95% CI), with *p* < 0.05 considered statistically significant. Where case data were missing, all available data were included in analyses; GEE is a statistical method for longitudinal analyses that is fairly robust for missing data [22]. All analyses were performed using SPSS version 27 (IBM, New York).

## Results

### Last T1D healthcare visit

Of 6338 young people (male 52.6%), 4877 (77.0%) attended paediatric diabetes centres. Mean age was 18.4 ± 2.3 years, age at T1D diagnosis 9.2 ± 4.4 years and T1D duration 8.7 ± 4.7 years. Most young people were born in Australia or its territories (85.6%), or New Zealand (5.4%), with 56 (1.4%) identifying as Aboriginal and/or Torres Strait Islander, and 63 (1.6%) as Māori. Treatment was with BD injections in 8.5%, MDI in 53.8% and CSII in 37.6%.

HbA1c was available in 5201 (82.1%) with mean 8.8 ± 1.9% (72.8 ± 68 mmol/mol). Overall, 2812 (44.4%) young people with T1D had their BP recorded; of these 840 (29.9%) had a systolic and/or diastolic BP in the hypertensive range. BMI was in the overweight or obese range in 1063/2527 (42.1%).

The characteristics of those who had their BP recorded at their last T1D healthcare visit are shown in Table 1, stratified by age (< 18 and ≥ 18 years). People with T1D aged ≥ 18 years who had their BP recorded (vs. those that did not) had a longer T1D duration (n = 1821, 10.2 ± 4.9 years vs. n = 1758, 9.4 ± 4.8 years; *p* < 0.001).

**Table 1:** Characteristics of youth in the ADDN registry who had a BP measurement recorded at their last T1D healthcare visit (between 2011 and 2020)

	Age < 18 years	Age ≥ 18 years	*p* value
n =	991	1821	
Age (years)	16.6 ± 0.5	20.0 ± 2.1	–
Sex: Male	529 (53.4)	928 (51.0)	–
T1D duration (years)	7.4 ± 4.1	10.2 ± 4.9	**< 0.001**
Systolic BP	0.3 ± 1.2	120 ± 13	–
In hypertensive range	122 (12.3)	432 (23.7)	**0.03**
Diastolic BP	0.2 ± 0.9	72 ± 9	–
In hypertensive range	48 (4.8)	496 (27.3)	**0.03**
Systolic and/or diastolic in hypertensive range	144 (14.5)	696 (38.2)	**< 0.001**
n =	931	1683	
*Insulin therapy*			
BD	87 (9.3)	101 (6.0)	**< 0.001**
MDI	454 (48.8)	918 (54.5)	**0.01**
CSII	389 (41.8)	663 (39.4)	0.34
n =	937	1492	
HbA1c (%)	8.9 ± 1.9	8.8 ± 1.8	0.90
(mmol/mol)	73.9 ± 20.4	72.3 ± 19.7	–
n =	985	1542	
Overweight/obese	335 (34.0)	728 (47.2)	**< 0.001**

Bold values indicate statistical significance

Data are mean ± standard deviation or n (%)

BD = Twice daily injections; BP = Blood pressure; CSII = Continuous subcutaneous insulin infusion; and MDI = Multiple daily injections

^*^Not all young people had complete data

**Fig. 1:**
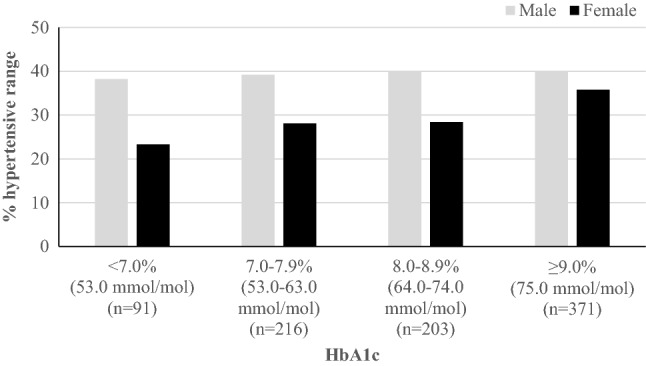
Any systolic and/or diastolic measurements in the hypertensive range at last diabetes healthcare visit (between 2011 and 2020), stratified by HbA1c and sex

Rates of hypertensive range BP measurements, regardless of age, stratified by HbA1c and sex are shown in Fig. 1. Greater proportions of males (vs. females) had a systolic and/or diastolic measurement in the hypertensive range, particularly in those categories < 9.0% (75 mmol/mol).

### All T1D healthcare visits

In people with T1D aged < 18 years, BP was recorded on 8573/21706 (39.5%) T1D healthcare visits. Of these, 1098 (12.8%) systolic, 352 (4.1%) diastolic, and 1267 (14.8%) of either systolic and/or diastolic BP measurements were in the hypertensive range; 246 (19.4%) met criteria for hypertension on ≥ 3 occasions. In multivariable GEE that included all visits, BP in the hypertensive range was associated with male sex, BD/MDI injection (vs. CSII) therapy, higher HbA1c and higher BMI SDS (Table 2).

**Table 2:** Multivariable generalised estimating equation modelling of systolic and/or diastolic blood pressure in the hypertensive range in youth in the Australasian Diabetes Data Network registry, recorded at T1D healthcare visits (between 2011 and 2020)

	> 95% percentile (< 18 years)^†^			≥ 130/80 mmHg (≥ 18 years)^‡^		
	B	95% CI	*p* value	B	95% CI	*p* value
T1D duration	0.01	− 0.01 to 0.02	0.47	− 0.001	− 0.02 to 0.02	0.94
Male vs. Female	0.41	0.28–0.56	**< 0.001**	0.53	0.36–0.70	**< 0.001**
BD/MDI vs. CSII	0.17	0.04–0.30	**0.01**	0.12	− 0.05 to 0.30	**< 0.001**
HbA1c	0.12	0.09–0.16	**< 0.001**	0.05	0.001–0.09	**0.02**
BMI SDS	0.43	0.35–0.51	**< 0.001**	–	–	
BMI	–	–	–	0.08	0.07–0.10	**< 0.001**

Bold values indicate statistical significance

T1D = Type 1 diabetes; BD = Twice daily injections; MDI = Multiple daily injections; CSII = Continuous subcutaneous insulin infusion therapy; and BMI = Body mass index

^†^n = 7351; ^‡^n = 4674

In those aged ≥ 18 years, BP was recorded on 7865/14949 (52.6%) T1D healthcare visits. Of these, 1618 (20.6%) systolic, 2190 (27.9%) diastolic, and 2901 (36.9%) of either systolic and/or diastolic BP measurements were in the hypertensive range; 630 (21.7%) met criteria for hypertension on ≥ 3 occasions. In multivariable GEE modelling, BP in the hypertensive range was again associated with male sex, BD/MDI injection (vs. CSII) therapy, higher HbA1c, and higher BMI (Table 2).

## Discussion

In this analysis of 6338 young people with T1D in Australasia, elevated BP was common and was in the hypertensive range in 19.4% of youth aged < 18 years and 21.7% of those aged ≥ 18 years. Risk factors for hypertension were male sex, BD/MDI injection therapy, higher HbA1c, and higher BMI. Modifiable risk factors and elevated BP start early. Findings flag the additive contribution of hypertension to the well-established body of evidence indicating a need to review healthcare models for adolescents and young adults with T1D.

The proportion of adolescents and young adults reporting BP in hypertensive ranges is cause for concern. Other data reported from ADDN [12], and smaller studies have confirmed this [10, 23–26]; one Australian study found up to 16% of adolescents with T1D had hypertension [10]. Further, a systematic review demonstrated hypertension was present in almost one in two young adults with T1D, although some of these studies applied out-dated diagnostic thresholds [5]. Altogether, these data indicate a pressing need to improve management and treatment to target levels in this vulnerable population.

Risk factors for hypertension were male sex, BD/MDI injection (vs. CSII) therapy, higher HbA1c, and higher BMI. Our findings are consistent with international data. For example, in a longitudinal adolescent cohort, boys have been found more likely than girls to develop high systolic BP as they approach adulthood [18]. Also, more than two decades ago the landmark Diabetes Control and Complications Trial demonstrated similar rates of hypertension amongst participants assigned to intensive vs. conventional insulin therapy [19]. However, the follow-up Epidemiology of Diabetes Interventions and Complications study found that intensive therapy reduced incident hypertension risk by 24% (hazard ratio = 0.76; 95% CI 0.64–0.92) [20]. Similarly, in a Brazilian study involving children and adolescents, elevated HbA1c was associated with increased BP [21]. Recent analyses of ADDN data involving children, adolescents and young adults [27, 28], in addition to findings from elsewhere in Australia [11, 29], have indicated persistently elevated HbA1c across-these age ranges. Finally, when considering higher BMI, the SEARCH for diabetes in youth study found that for each 0.01 unit of annual increase in waist‐to‐height ratio of youths with T1D, the adjusted relative risk for hypertension was 1.53 (95% CI 1.36–1.73) [30].

BP was only routinely recorded in 39.5% of visits by youth aged < 18 years, and 52.6% of visits by those aged ≥ 18 years. Australian national evidence-based clinical care guidelines for T1D in children, adolescents and adults [9], and guidelines published by the American Diabetes Association (ADA) and International Society for Pediatric and Adolescent Diabetes (ISPAD) [7, 31] all recommend annual BP screening. While we did not determine the frequency of BP recorded amongst individual young people with T1D, in view of the persistently elevated BP values discovered, the priority is both to ensure universal monitoring, even in younger-age adolescents, and for hypertension to be followed up to ensure treatment to target.

Our findings have some limitations. Firstly, some data were incomplete, which is a recognised issue with registry data. Missing data may reflect clinicians or centres that do not routinely measure BP. However, given the sample size of the study population, this is not likely to result in lack of generalizability of our findings. We acknowledge the possibility of selection bias in those who with higher BP at the time of initial measurement, or those with risk factors (such as being overweight or family history) may have been more likely to have it repeated. Further, a diagnosis of hypertension currently requires BP measurements from more than one occasion [7, 9, 31]. Our data did not report confirmed diagnoses of hypertension, only the incidence, associated variables and associations with elevated BP. We had no access to data relating to presence of proteinuria, which would have indicated where lower systolic and diastolic BP targets were appropriate [32], or to use of anti-hypertensives. It may be that some of the normal range BP measurements reflected effective pharmacological management. Data were also not available for many important socioeconomic characteristics. As the ADDN database expands, with more detailed data examining the association of BP, pharmaceutical treatment and association with other key outcomes, a more complete and representative record of BP for this population is anticipated. Finally, hypertension diagnosis and management can be complicated by masked hypertension and white-coat hypertension. This is in addition to BP measurement technique, use of manual sphygmanomter’s versus electronic devices, and timing of measurements; aspects that were not accounted for.

A major strength of this research is the sample size of available BP measurements and related data. ADDN data are predominantly derived from tertiary hospital diabetes clinics where the majority of young people are managed, while young adults are seen in diabetes clinics in both the public and private sector, as well as by general practitioners [33]. Nevertheless, the size and scope of this database suggests that data are reasonably representative and were analysed using methods (multivariable GEE modelling) widely recognised as robust.

In conclusion, this study demonstrates hypertensive BP measurements are unacceptably high in adolescents and young adults, occurring from young ages. Findings flag the additive contribution of hypertension to the well-established body of evidence indicating a need to review healthcare models for adolescents and young adults with T1D. Targeting modifiable risk factors such as glycaemic control and overweight/obesity may lead to a reduction in the burden of hypertension and its potential long-term morbidity and mortality in this population.
